# TwinFedPot: Honeypot Intelligence Distillation into Digital Twin for Persistent Smart Traffic Security

**DOI:** 10.3390/s25154725

**Published:** 2025-07-31

**Authors:** Yesin Sahraoui, Abdessalam Mohammed Hadjkouider, Chaker Abdelaziz Kerrache, Carlos T. Calafate

**Affiliations:** 1 Intelligent Systems Engineering Department (ISE), National Higher School of Artificial Intelligence (ENSIA), Algiers 16000, Algeria; 2 Department of Computer Science and Information Technology, University of Ouargla, Ouargla 30000, Algeria; 3 Laboratoire d’Informatique et de Mathématiques, Université Amar Telidji de Laghouat, Laghouat 03000, Algeria; ch.kerrache@lagh-univ.dz; 4 Computer Engineering Department (DISCA), Universitat Politècnica de València, 46022 Valencia, Spain

**Keywords:** digital twin, honeypots, federated distillation, Zero-Shot Learning, cybersecurity, Internet of Vehicles (IoV)

## Abstract

The integration of digital twins (DTs) with intelligent traffic systems (ITSs) holds strong potential for improving real-time management in smart cities. However, securing digital twins remains a significant challenge due to the dynamic and adversarial nature of cyber–physical environments. In this work, we propose TwinFedPot, an innovative digital twin-based security architecture that combines honeypot-driven data collection with Zero-Shot Learning (ZSL) for robust and adaptive cyber threat detection without requiring prior sampling. The framework leverages Inverse Federated Distillation (IFD) to train the DT server, where edge-deployed honeypots generate semantic predictions of anomalous behavior and upload soft logits instead of raw data. Unlike conventional federated approaches, TwinFedPot reverses the typical knowledge flow by distilling collective intelligence from the honeypots into a central teacher model hosted on the DT. This inversion allows the system to learn generalized attack patterns using only limited data, while preserving privacy and enhancing robustness. Experimental results demonstrate significant improvements in accuracy and F1-score, establishing TwinFedPot as a scalable and effective defense solution for smart traffic infrastructures.

## 1. Introduction

In recent years, the proliferation of advanced technologies has revolutionized various aspects of daily life, with the Internet of Vehicles (IoV) emerging as a transformative innovation in modern transportation systems. IoV leverages built-in sensors and advanced communication technologies to facilitate real-time data exchange among vehicles, surrounding environments, and other smart devices [1,2]. This capability reshapes the future of smart cities by improving traffic efficiency and fostering a seamless, interconnected ecosystem. Through IoV, vehicles can communicate and share data not only with each other but also with a wide array of smart devices, enabling a highly intelligent and adaptive transportation network. This interconnectedness promotes efficient traffic management, reduces congestion, and supports sustainable urban development. Furthermore, IoV applications extend across various fields, including environmental monitoring, public safety, and urban planning [3], contributing to the development of safer, more efficient, and environmentally friendly urban environments.

However, the accelerating pace of urbanization and the increasing vehicle density pose a significant challenge to contemporary urban planners, particularly in tackling traffic congestion. To address this issue, DT technology has emerged as an innovative solution for managing and optimizing traffic flow [4]. By enabling real-time simulation, analysis, and decision-making, DTs enhance intelligent traffic management and pave the way for more efficient and sustainable urban living. The DT market is anticipated to experience substantial growth in the coming years, driven by its transformative potential in traffic management and urban mobility. This advanced technology provides real-time insights to authorities, facilitating a comprehensive and timely understanding of traffic dynamics. By harnessing historical datasets and leveraging AI-driven predictive analytics, DT systems can predict traffic patterns accurately, enabling proactive measures to mitigate congestion and enhance road safety. Moreover, DT technology plays a crucial role in optimizing responses to planned and unplanned disruptions [5], including special events, road closures, and emergencies, thus ensuring smoother traffic flow and enhancing the efficiency and resilience of transportation systems.

While DT systems are increasingly integrated into ITS infrastructures, driving advancements in traffic management, they also broaden the attack surface. This causes an increasing exposure to cyber threats, which has led to a significant rise in cyberattacks targeting traffic management ecosystems. Consequently, the transportation sector has become one of the most frequently targeted domains, with each cyber incident costing an estimated USD 3.58 million in 2020 [6]. Although various security mechanisms, such as cryptography, firewalls, blockchain-based solutions, and Federated Learning (FL), have been applied to secure DTs, each presents limitations that hinder their effectiveness in this context. Cryptographic techniques and firewalls often lack real-time adaptability, and they struggle to scale in highly dynamic environments. Blockchain approaches face challenges, including limited scalability, high energy consumption, and transaction latency [7], which compromise their practicality for real-time operations. FL, while promising for privacy preservation, introduces vulnerabilities such as model poisoning, due to the sharing of complete model architectures and updates. As a result, these limitations leave DTs exposed to evolving cyber threats, particularly insider attacks that can evade detection within complex, interconnected systems.

In this study, we address the limitations of traditional FL by introducing TwinFedPot, a novel inverse distillation framework that integrates honeypots as semantic sensing units within the DT framework. Instead of training models locally, honeypots capture environment-specific cyber threats, and generate semantic representations using ZSL models. They then distill the insights to the DT server, which aggregates the soft knowledge, and continually refines a global teacher model in a privacy-preserving manner. This inversion of the classical FD paradigm not only eliminates client-side training overhead, but also enables the detection of novel or evolving attack vectors without the need for labeled data at the edge. Our system is designed specifically for smart traffic management infrastructures in smart cities, where real-time responsiveness, scalability, and robustness are essential.

The key contributions of this work are as follows:

- We introduce a novel inverse distillation strategy where honeypots act as passive learners and generate semantic logits using Zero-Shot Learning, rather than training local models.
- We implement TwinFedPot, a DT-based framework that aggregates logits at the central server to construct and update a teacher model capable of real-time threat classification without raw data sharing.
- Our approach enhances system scalability, adaptability, and privacy, making it suitable for large-scale, real-time smart traffic infrastructures and other cyber–physical systems.
- Extensive experiments on the CIC-IoT2023 dataset validate our system’s performance, showing improved generalization to unseen attacks, and reduced computational burden at the edge.

The rest of this paper is organized as follows: in Section 2, we review some related works. Then, Section 3 details the proposed model. Extensive experiments are presented in Section 4. Finally, we conclude this paper in Section 5, and refer to future work.

## 2. Literature Review

In this section, we review the existing literature on digital twins, honeypots, and federated distillation. Each concept is introduced and discussed in detail in the following subsections.

### 2.1. Digital Twins

**Figure 1:**
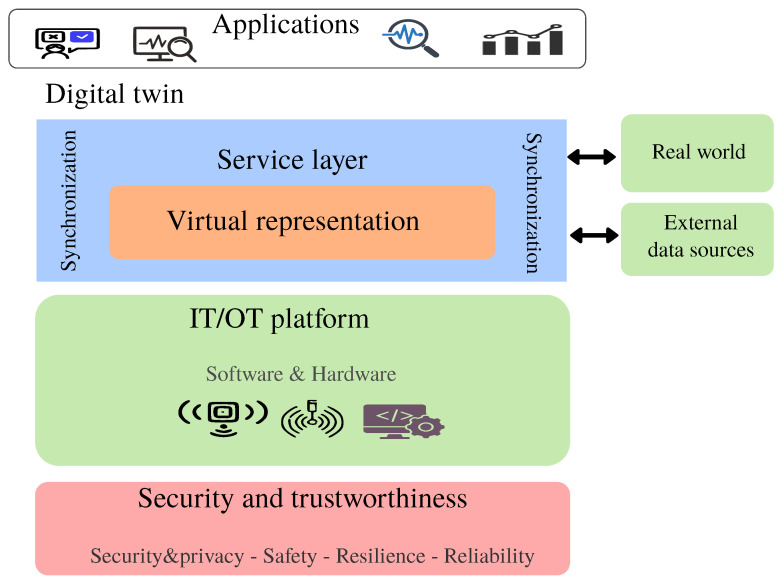
DT platform stack architectural framework.

Digital twins are dynamic digital counterparts of physical assets, systems, or processes, and play a foundational role in Industry 4.0. They are generally classified into three categories: (i) digital models, which are static and updated manually or not at all; (ii) digital shadows, which receive one-way updates from the physical system; and (iii) digital twins, characterized by bidirectional synchronization [4]. While only the latter meets the strict DT definition, all three fall under the broader DT paradigm. These systems integrate real-time sensor data, simulation techniques, mathematical modeling, 3D scans, and ML to construct accurate, real-time representations of their physical counterparts. As illustrated in Figure 1, DT architectures support layered integration with external data sources, enabling high-fidelity monitoring, improved decision-making, and enhanced sustainability.

Moreover, DT systems, composed of multiple coordinated DT instances [8], represent a cornerstone of contemporary technological advancements. The Architectural Framework for DT Platform Stack Architecture (PSAF), promoted by the DT Consortium [9], provides a structured reference for developing secure and scalable DT systems. As shown in Figure 1, it comprises the following core layers:

- IT/OT infrastructure: This layer connects the physical and digital environments using networks, sensors, cloud platforms, and computing resources. It ensures reliable data flow, system scalability, and operational efficiency.
- Virtual representation: This component models the physical system using methods such as 3D modeling, simulation, or ML. It is continuously updated to reflect real-world conditions accurately.
- Service layer: Acting as middleware, this layer provides functions for accessing, updating, and synchronizing digital twins. It maintains consistency through one-way, two-way, or real-time data exchange.
- Security and trustworthiness: This layer ensures data integrity, access control, and transparency. It defines roles, responsibilities, and policies to protect the system from cyber threats.
- Applications: This top layer focuses on real-world implementations across domains such as healthcare, smart manufacturing, energy, and urban planning, showcasing the value of DTs in improving decision-making and performance.

Although DTs offer significant benefits in system monitoring and optimization, their integration with real-time data and networked environments raises concerns regarding security, privacy, and reliability, necessitating robust safeguards across the entire stack.

### 2.2. Honeypots

Honeypots in cybersecurity are decoy systems designed to attract attackers, allowing security analysts to study their behavior while protecting real assets. They fall into two categories: (i) static honeypots, which are simpler but more easily detected, and (ii) dynamic honeypots, which use adaptable configurations to better deceive intruders and analyze their tactics [10,11].

Based on the above classification, Shi et al. [11] investigated a dynamic distributed honeypot system that integrates private blockchain, encrypted communication, and service transformation to confuse intruders. The security of the proposed design is validated through both theoretical analysis and practical attack simulation. The authors in [12] proposed AI-driven adaptive honeypots in the cloud that dynamically adapt configurations using real-time threat intelligence, while providing deeper insight into attacker behavior and improving defense strategies. Experiments confirm their greater effectiveness in evolving threat landscapes. Wang et al. [13] developed a cooperative defense framework for UAVs using game theory. It creates a system where UAVs share honeypot data, supported by incentive mechanisms and fair contracts to prevent free-riding and encourage participation. Through advanced learning techniques and simulations, the framework enhances UAV performance and strengthens defenses in dynamic cybersecurity environments. Wang et al. [14] leveraged large language models (LLMs) and prompt-based engineering techniques to design and deploy an adaptive honeypot framework named honeypotGPT, addressing their traditional limitation of being unable to respond proactively to attackers adopting evolving tactics. Their experimental results demonstrated the superior effectiveness and efficiency of the proposed solution.

### 2.3. Federated Distillation (FD)

FD is a decentralized learning paradigm derived from knowledge distillation, where multiple student models collaboratively train a central model (teacher) by sharing soft outputs (logits or class probabilities) instead of raw data [15]. This approach is particularly effective when data privacy is crucial, such as in network security systems. Each student computes a temperature-scaled softmax over its local model logits $z=[z_{1},z_{2},\ldots ,z_{K}]$ to generate softened class probabilities:

(1) $$P_{i}=\frac{\exp(z_{i}/T)}{\sum_{j=1}^{K}\exp\left(z_{j}/T\right)}$$

Several methods are commonly used to aggregate knowledge at the teacher server:

- Simple voting: In this approach, each student model transmits its hard class prediction (i.e., the argmax of its output probability distribution) to the teacher. The teacher then aggregates these predictions and selects the most frequently occurring class label among them. For example, the authors of [16] use the majority vote of lightweight student models to determine the final image classification output.
- Logit averaging: Students send raw logits or softmax outputs, which are averaged to form a consensus. This approach has been effectively adopted in various domains. For instance, Sahraoui et al. [15] designed a federated distillation framework in the IoV-healthcare context, where the server broadcasts influenza-related insights, and clients return distilled logits, ensuring privacy and reducing communication overhead.
- Kullback–Leibler (KL) divergence: The teacher receives soft probability distributions (obtained by applying softmax to logits) from the student models and computes their average to form a consensus distribution. The teacher model is then updated by minimizing the KL divergence between its own predicted probability distribution and this aggregated consensus. The authors of [17] refer to this loss as “oracle loss”, which aligns the teacher’s output with the collective semantic knowledge distilled from the students.

### 2.4. Related Works

To enhance the security of DT, the literature presents a wide range of methods and approaches. In this section, we classify the most relevant contributions based on their underlying enabling technologies.

#### 2.4.1. Blockchain-Based Security Frameworks

Several works have introduced blockchain technologies to enhance data integrity, transparency, and authentication in DT environments. For instance, Putz et al. [18] proposed Ethertwin, a blockchain- and smart contract-based system that secured DT data and interactions. It ensured integrity, transparency, and decentralized trust among stakeholders without a central authority. While offering strong security, it suffered from performance overhead and integration challenges in real-time environments. The work in [19] introduced a secure DT platform using blockchain to help small manufacturers offer their resources as digital services. It included a smart system to match services between providers and users. The platform ensured trust and traceability in all manufacturing activities. The authors of [20] presented a secure DT-enabled Industrial IoT architecture, using edge and cloud blockchain layers for trusted data exchange, and deep learning for intrusion detection. Smart contracts and off-chain encrypted storage ensured privacy and efficiency, while LSTM-based models detected cyber threats effectively. In the healthcare domain, the authors of [21] introduced a blockchain-secure patient DT to protect personal health data and manage secure sharing using smart contracts. They introduced a lightweight cryptographic scheme using a permissioned blockchain to ensure privacy and verify data origin. The system enabled trusted, automated DT updates and interactions in healthcare. Kumar et al. [22] proposed a robust cybersecurity framework that integrated explainable artificial intelligence (XAI) with smart contracts to enable a blockchain-based authentication scheme, ensuring reliable data acquisition. A DT incorporating various attack scenarios was developed to evaluate an intrusion detection system (IDS) within a Zero Trust Network (ZTN) environment. Experimental results demonstrated high accuracy across security metrics. However, a key limitation lies in the framework’s reliance on specific datasets, which may limit its applicability to different environments. In the same context, Khan et al. [23] introduced TwinChain, a blockchain-based framework designed to resist quantum attacks, enable instant transaction confirmation, reduce operational costs, and secure DT transactions with an emphasis on both efficiency and security. The framework’s effectiveness was demonstrated through a case study involving surgical robot manufacturing. Lu et al. [24] proposed a DT wireless network (DTWN) model where end users were associated with DTs at base stations. To secure DT instances against untrusted users and enhance edge intelligence, they introduced a permissioned blockchain-based FL scheme. The model further optimized resource allocation and reduced latency using multi-agent reinforcement learning. Liao et al. [25] proposed a blockchain-enabled DT as a Service (DTaaS) model for ITS. A double auction algorithm was used for matching DT providers with requesters based on demand. To ensure secure and efficient transactions, a new DT-Delegated Proof of Stake (DT-DPoS) consensus mechanism was introduced.

#### 2.4.2. ML/DL-Based Security Architectures

Several efforts utilize machine learning (ML) and deep learning (DL) to secure digital systems. Mikail et al. [26] presented a threats detection framework combining DT and FL to identify zero-day attacks in IoT healthcare networks. The framework introduced an Adaptive Thresholding with Early Stopping (ATES) mechanism to enhance model training efficiency and reduce CPU overhead. Experimental results demonstrated superior performance in terms of detection accuracy, latency, and resource efficiency compared to traditional federated averaging methods. Krichnaveni et al. [27] proposed TwinSec-IDS, a smart security system for industrial networks to protect digital twins. It uses a set of deep learning models, and selects the most relevant features to detect cyberattacks with high accuracy. The system is able to enhance real-time protection and improve the overall safety of industrial processes. The authors in [28] presented a Cloud-Based DT framework to simulate IT infrastructures and predict cyber threats in real time. It leverages traditional ML models (Random Forest, CNN, LSTM) to enhance detection accuracy, with LSTM achieving 96.1%. This approach offers scalable, automated, and proactive cybersecurity defense via cloud deployment. Suhail et al. [29] leveraged connected vehicles and crowdsourced applications to enhance the security assessment and training capabilities of DTs in cyber–physical systems (CPSs). Their approach provides real-time data streams and establishes a dynamic environment for gamification-based security training, enabling analysts to identify vulnerabilities, simulate attack scenarios, and improve decision-making using explainable AI (XAI) models. The integration of gamification offers an interactive platform for testing and refining cybersecurity strategies in a realistic and engaging manner. Yigit et al. [30] developed a DT-enabled framework for DDoS detection in Internet Service Provider (ISP) core networks, combining online learning, automated feature selection, and Multilayer Perceptron (MLP) for classification, achieving 97% accuracy. However, the framework is limited to DDoS attack detection, failing to address other types of cyber threats. The same authors [31] proposed TwinPot, a DT-assisted honeypot framework for external and internal attack detection in smart seaports, employing an Automated Classification Method (AutoCM). However, its reliance on pre-labeled data, domain-specific validation, and limited adaptability to dynamic threats constrains its generalizability and robustness (See Table 1 for a detailed comparison).

## 3. System Model

**Figure 2:**
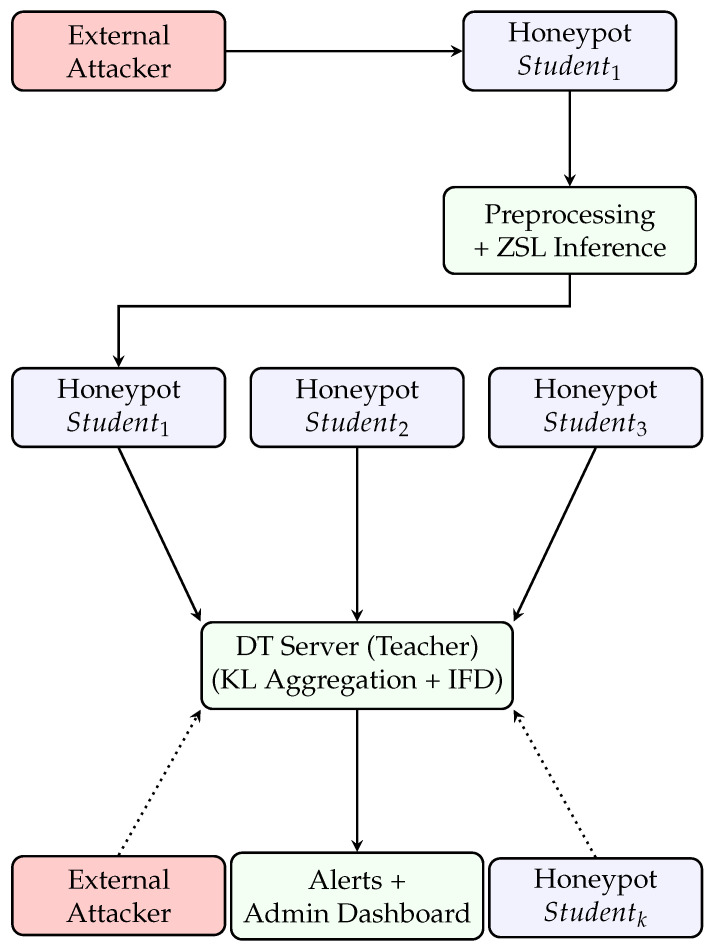
Data flow in TwinFedPot framework.

As illustrated in Figure 2, this section introduces TwinFedPot, a DT framework designed for real-time traffic management and cybersecurity. TwinFedPot integrates distributed honeypots with IFD learning scheme, enabling collaborative threat detection while preserving data privacy and minimizing communication overhead.

We define the components of our proposed TwinFedPot framework as follows:

- **$H=\{h_{1},\ldots ,h_{K}\}$**: A set of distributed honeypots deployed across the network.
- **$D_{k}$**: The local dataset at honeypot $h_{k}$, consisting of samples $\left(x_{i}^{k},y_{i}^{k}\right)$, where each $x_{i}^{k}$ is a log-derived feature vector continuously collected from system activities.
- **$y_{i}^{k}$**: Labels annotated using the MITRE ATT&CK framework [32], representing observed Tactics, Techniques, and Procedures (TTPs).
- **$f_{k}$**: A local student model at honeypot $h_{k}$, trained or fine-tuned on dataset $D_{k}$, or used to infer unseen attack classes using ZSL.
- **$f_{t}$**: A global teacher model hosted on the DT cloud server, updated using knowledge distilled from the honeypots.
- **$P_{k}\left(x\right)$**: The soft prediction (logits or probability vector) produced by the student model $f_{k}$ for input *x*.

TwinFedPot employs IKD based on Kullback–Leibler (KL) divergence. Each honeypot either trains locally or performs zero-shot inference via semantic similarity, and communicates only its soft predictions $P_{k}\left(x\right)$ to the DT server, preserving data privacy by avoiding the transmission of raw inputs or model weights.

The DT aggregates these soft predictions as follows:

(2) $$\bar{P}\left(x\right)=\frac{1}{K}\sum_{k=1}^{K}P_{k}\left(x\right)$$

This aggregated distribution is then used to update the teacher model $f_{t}$ by minimizing the KL divergence:

(3) $$\min_{f_{t}}\sum_{x}D_{\mathrm{KL}}\left(\bar{P}\left(x\right)∥f_{t}\left(x\right)\right)$$

where $D_{\mathrm{KL}}\left(\bar{P}\left(x\right)∥f_{t}\left(x\right)\right)$ measures the divergence between the students’ consensus prediction and the teacher’s output. The distillation loss is formally defined as

(4) $$L_{FD}=\sum_{i=1}^{K}P_{i}^{t}\log\left(\frac{P_{i}^{t}}{P_{i}^{s}}\right)=-\sum_{i=1}^{K}P_{i}^{t}\log P_{i}^{s}+\sum_{i=1}^{K}P_{i}^{t}\log P_{i}^{t}$$

Here, $P_{i}^{t}$ denotes the aggregated soft label from the students, and $P_{i}^{s}$ is the prediction of the teacher model $f_{t}$ on the input $x_{i}$.

This inverse distillation strategy allows the DT to learn from the distributed knowledge of honeypots without requiring access to raw traffic data. The data flow in the proposed security framework (see Figure 2) captures the interaction between attackers, honeypots, and the DT server using ZSL and IFD.

TwinFedPot offers key advantages, including strong privacy preservation—since raw logs and model parameters remain local—and efficient, scalable learning across decentralized honeypots without direct data or weight sharing.

The architecture operates through two communication layers: (i) the Physical-to-Virtual (P2V) layer, which ensures real-time synchronization between the physical infrastructure and its digital counterpart; and (ii) the Virtual-to-Virtual (V2V) layer, which enables a learning-driven traffic DT system, reinforced by an adaptive honeypot network for robust cybersecurity.

The TwinFedPot ecosystem consists of three interdependent components:

- Physical assets: Each vehicle is equipped with a Telematics Control Unit (TCU), an embedded device that collects data from key vehicle sensors (e.g., GPS, OBD-II, fuel level, tire pressure, speed). The TCU processes these real-time data and transmits them to a cloud server via wireless networks (e.g., GPRS, cellular, LTE) in a structured format for further analysis.
- Traffic digital twin (TDT) system: Hosted on a cloud server, the TDT continuously processes and analyzes incoming data (as outlined in Algorithm 1) using advanced algorithms and ML techniques. This processing yields actionable, real-time insights for congestion analysis, dynamic route optimization, and predictive navigation. The system also integrates the global FD model, which aggregates the distilled knowledge from local honeypots. This aggregation mechanism functions as a decay strategy, mitigating potential attacks targeting the physical infrastructure and enhancing overall system resilience.

**Algorithm 1** TwinFedPot: inverse FD for DT**Require:** Initial DT model parameters $\theta^{g(0)}$, temperature scaling factor *T***Ensure:** Updated global DT model $\theta^{g}$  1: **while** any honeypot $h_{k}\in H$ is active **do**  2:       **for** each honeypot $h_{k}$ in parallel **do**  3:             Acquire and preprocess local log data from host system:$$D_{k}\leftarrow \mathrm{ExtractLogFeatures}\left(\right)$$
                    ▹*Logs are parsed into structured input features, preserving on-device data privacy*.  4:             Apply the local zero-shot student model $f_{k}$ to each input $x\in D_{k}$  5:             Compute soft predictions using temperature-scaled softmax:$$P_{k}\left(x\right)=\mathrm{Softmax}\left(\frac{f_{k}\left(x\right)}{T}\right)$$
                          ▹*Distilled logits capture nuanced class likelihoods for downstream aggregation.*  6:             Transmit $P_{k}\left(x\right)$ to the DT server       ▹ *Only soft labels are shared.*  7:       **end for**  8:       Aggregate all received client predictions:$$\bar{P}\left(x\right)=\frac{1}{K}\sum_{k=1}^{K}P_{k}\left(x\right),\;\forall x\in D$$                              ▹*Aggregated knowledge captures a consensus view across honeypots.*  9:       Update the DT model $f_{t}$ by minimizing the KL-based distillation loss:$$\theta^{g(t+1)}\leftarrow \theta^{g(t)}-\eta \cdot \nabla_{\theta }\left(\sum_{x\in D}L_{\mathrm{KD}}\left(f_{t}\left(x\right),\bar{P}\left(x\right)\right)\right)$$
         ▹*Distillation Process: the DT learns to mimic the clients’ averaged logits via KL divergence.*  10:       Optionally broadcast updated teacher predictions to honeypots.  11:       Increment round: t←t+1  12: **end while**
          **return** Continuously enhance DT model $\theta^{g}$       ▹ *TwinFedPot provides scalable, privacy-preserving, and attack-aware without training and raw data sharing.*

**Figure 3:**
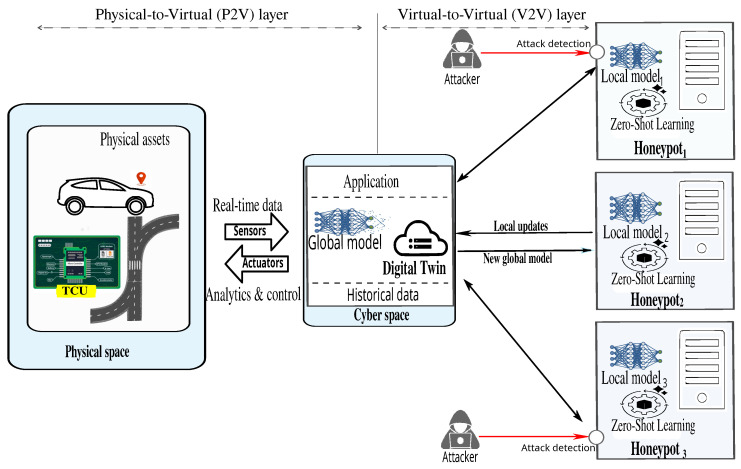
TwinFedPot framework.

- Adaptive honeypot network: Cloud-based honeypots are strategically deployed across multiple servers, leveraging high-speed monitoring and logging to detect and record malicious activities in real time. The intelligent detection process unfolds through several key stages:
  - Attack detection and logging: Each honeypot mimics a genuine system, enticing attackers to interact with it. Upon an attempted intrusion, the honeypot logs detailed information such as
    * Source of the attack (IP address, geolocation, attack vector);
    * Command execution logs;
    * Traffic patterns and anomalies;
    * Access attempts and privilege escalation;
    * Protocols used (e.g., TCP, UDP, ICMP).
  Before local model training or ZSL-based inference, raw attack data are preprocessed to extract meaningful features, such as access frequency, request patterns, and behavioral anomalies, and to label known attacks based on observed behavior.
  - Local model training/inferencing: Each honeypot either trains a local model or ZSL on its processed log data. By analyzing features like access frequency and behavioral patterns, the honeypot identifies both known and novel attacks, enabling adaptive detection without prior labeled examples.
  - IFD for global defense: Each honeypot shares distilled knowledge (e.g, logits or soft labels) or updated model parameters with the central server. This secure knowledge exchange strengthens the global model’s ability to detect emerging and previously unseen threats while maintaining low communication overhead and preserving data sovereignty. As illustrated in Figure 3 and formalized in Equations (2) and (3), the DT aggregates these soft predictions and refines the global model through KL-divergence minimization, enabling robust and generalized threat detection.

## 4. Evaluation Analysis

This section presents the evaluation of the proposed TwinFedPot defense mechanism, designed to enhance intrusion detection in honeypot-based environments. TwinFedPot operates within the TDT system, where a global model is collaboratively updated through IFD. The evaluation involves multi-class classification, treating each attack type as a distinct class.

To rigorously evaluate the effectiveness of the proposed design, we use the following key performance metrics:

- Accuracy, representing the proportion of correctly classified instances, is given by
  (5) $$\mathrm{Accuracy}=\frac{TP+TN}{TP+TN+FP+FN}$$
  where TP denotes true positives, TN true negatives, FP false positives, and FN false negatives.
- True Positive Rate (TPR), also referred as recall or sensitivity, measures the fraction of actual attacks that are correctly identified:
  (6) $$\mathrm{TPR}=\frac{TP}{TP+FN}$$
- False Positive Rate (FPR) quantifies the proportion of benign instances misclassified as attacks, calculated as
  (7) $$\mathrm{FPR}=\frac{FP}{FP+TN}$$
- F1-score, which provides a harmonic mean between precision and recall, is defined as
  (8) $$F1-\mathrm{score}=2\times \frac{\mathrm{Precision}\times \mathrm{Recall}}{\mathrm{Precision}+\mathrm{Recall}}$$
  where Precision is computed as
  (9) $$\mathrm{Precision}=\frac{TP}{TP+FP}$$

To comprehensively evaluate the performance of the proposed TwinFedPot framework, we benchmark it against several ML and deep learning (DL) algorithms, including Random Forest, Support Vector Machines, Dense Neural Networks, etc. These baselines are chosen for their proven effectiveness in intrusion detection and their ability to capture complex patterns. Implementation is carried out using Python-based libraries such as NumPy, Pandas, Scikit-learn, and TensorFlow/Keras, with SMOTE employed to mitigate class imbalance. Within TwinFedPot, student models employ ZSL to generate soft predictions from semantic embeddings, which are subsequently aggregated at the DT server via IFD. This experimental setup enables a fair comparison between conventional models and TwinFedPot, underscoring the latter’s strengths in scalability, generalization, and its ability to detect novel or low-frequency attack classes without the need for explicit labels.

### 4.1. Dataset

The effectiveness of the proposed TwinFedPot framework is validated using the CIC IoT2023 dataset [33], a comprehensive repository of labeled network traffic tailored for IoT security research. It consists of 46,686,579 recorded events and 47 distinct features, capturing 33 attack types organized into 7 major categories: Reconnaissance (Recon) Attacks, Distributed Denial-of-Service (DDoS) Attacks, Web-Based Attacks, Spoofing Attacks, Brute-Force Attacks, Mirai-Based Attacks, and Man-in-the-Middle (MitM) Attacks.

For experimentation, the dataset is strategically partitioned to either train local models at honeypot nodes or to support ZSL for inferring previously unseen attack classes. It includes critical network attributes such as traffic flow statistics, inter-arrival times, packet size distributions, and flag-based indicators, providing a rich and realistic foundation for developing scalable intrusion detection mechanisms across IoT environments.

The CIC IoT2023 dataset was selected for its extensive coverage of real-world IoT attack scenarios and well-labeled traffic patterns, which make it ideal for evaluating the proposed framework. While our validation is based on this dataset, the TwinFedPot framework is inherently dataset-agnostic, being designed to generalize across diverse cyber–physical system (CPS) environments.

### 4.2. Experimental Results

The experimental workflow begins with data preprocessing, where we isolate attack instances specifically associated with honeypot-based DT attack prevention. Certain attacks are more likely to target decoy systems through scanning, probing, or exploitation attempts. We focus on 4 high-impact threats, A = {DDoS-Slowloris, DNS Spoofing, SQL Injection, Cross-Site Scripting (XSS)}, as the primary threat categories. To improve computational efficiency and enhance model performance, we refine the dataset by including only these critical attack types.

**Figure 4:**
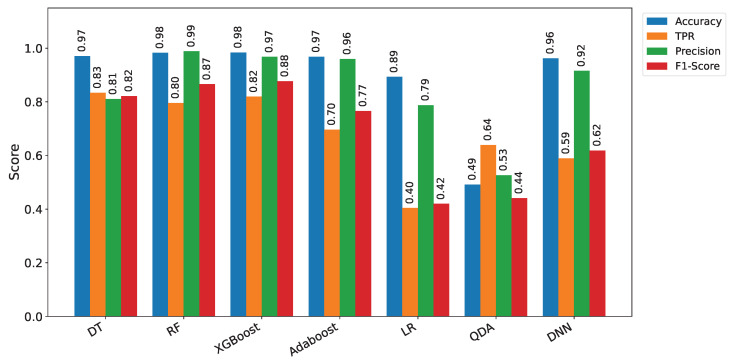
Honeypot ML models’ performance comparison.

TwinFedPot performs zero-shot inference on 64 network flow sentences in under 0.019 s using the all-MiniLM-L6-v2 model on a standard Colab CPU (Intel Xeon), eliminating the need for local training, and enabling efficient real-time detection. In parallel, Lightweight KL-based aggregation at the server further reduces computation and communication overhead, aligning with recent findings in efficient federated distillation [34] and zero-shot cost prediction [35]. We first evaluated traditional ML and DL models on the CIC IoT 2023 dataset, as illustrated in Figure 4 and detailed in Table 2. Classical models such as Random Forest and XGBoost achieved a notable high classification accuracy on the client side, up to 98%. However, despite these promising results, these models exhibit critical limitations. In particular, their true positive rates (TPRs) remain suboptimal (≤0.82), indicating a reduced ability to correctly detect actual attacks. In addition, these methods are prone to overfitting and impose significant computational overhead, making them less suitable for deployment in resource-constrained IoT environments.

**Figure 5:**
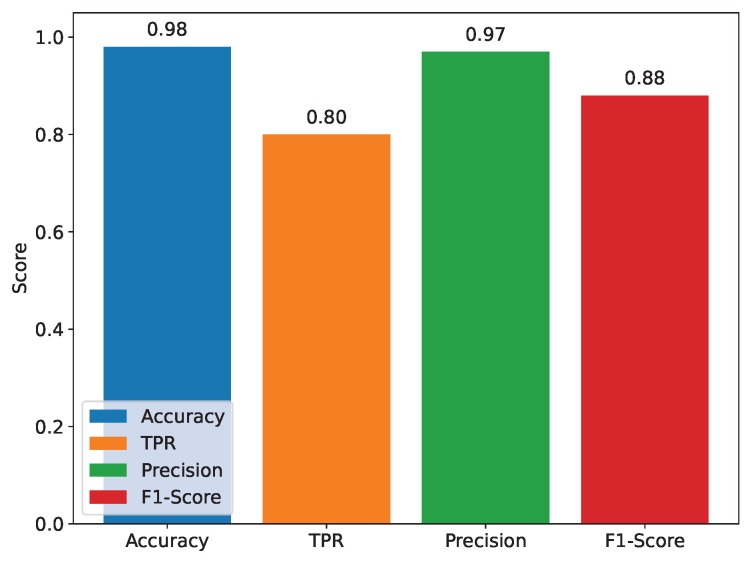
Teacher model performance using XGBoost.

Furthermore, while the centralized DT server achieved similar high accuracy levels, as shown in Figure 5, reliance on centralized training introduces concerns about data privacy, communication latency, and limited scalability, due to the need to share raw data between honeypot nodes.

To overcome these limitations, we propose an efficient threat detection framework grounded in ZSL using all-MiniLM-L6-v2 embeddings. Our approach eliminates the need for local model training at honeypot nodes by leveraging semantic representation techniques to directly map features to emerging threat instances. The DT server aggregates soft knowledge (logits) from honeypots using KL divergence-based distillation, facilitating robust generalization without direct data access.

**Figure 6:**
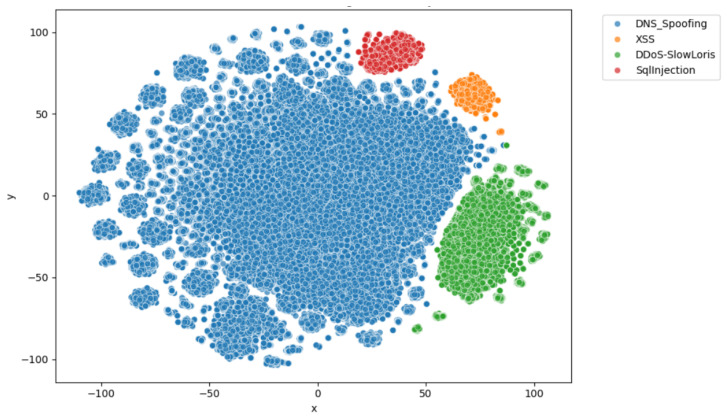
t-SNE visualization of zero-shot text embeddings.

Figure 6 presents a t-SNE projection of zero-shot text embeddings generated using the all-MiniLM-L6-v2 model, with points colored according to ground truth labels (DNS Spoofing, XSS, DDoS-SlowLoris, SqlInjection). The formation of distinct clusters demonstrates that the embeddings capture class-wise separability effectively, underscoring their suitability for supporting security analysis and classification tasks within our framework.

**Figure 7:**
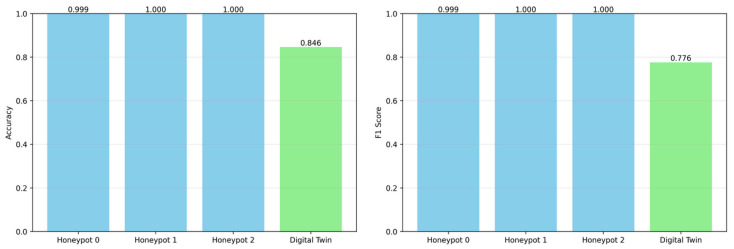
Accuracy and F1-score comparison: DT vs. honeypots.

Figure 7 illustrates the comparative performance of the three honeypots and the central DT model in terms of accuracy and F1-score, highlighting the effectiveness of the proposed IKD strategy. Each honeypot achieves near-perfect classification performance (accuracy and F1 ≈1.0), benefiting from environment-specific data and localized learning. In contrast, the DT exhibits slightly lower scores (accuracy = 0.846, F1 = 0.776), reflecting the challenge of generalizing across heterogeneous honeypot outputs. However, by minimizing the KL divergence between the predicted probability distributions of the honeypots and its own, the DT effectively aggregates soft knowledge. This approach enables it to approximate the semantic insights of individual honeypots without accessing raw data, thereby preserving both privacy and scalability. Overall, these results confirm that the KL-guided distillation process enables the DT to infer generalized attack patterns from distributed, zero-shot predictions, making the system both adaptive and robust for real-time threat detection.

## 5. Conclusions and Future Work

This paper introduced TwinFedPot, a DT-driven framework that combines zero-shot honeypot intelligence with IFD to address cybersecurity threats in smart traffic management systems. By distilling predictions from distributed clients into a centralized DT, our approach achieves high detection accuracy with minimal data requirements. The reverse distillation mechanism enhances the global model’s adaptability to unseen attacks while preserving local data privacy. TwinFedPot not only strengthens the security of digital twins in adversarial environments but also paves the way for scalable and interpretable CPS protection in future smart cities.

As future work, we plan to evaluate the framework on additional datasets to further demonstrate its generalizability. We also aim to integrate a permissioned blockchain system to validate and authenticate participating honeypots, thereby preventing unauthorized or malicious honeyposts from distorting the IFD process, or biasing the model outcomes.

## Figures and Tables

**Table 1:** Comparison of DT-based security approaches.

Study	Decentralized Arch.	Tech.	Attack Awareness	Privacy Support	Defense Adaptivity	Resource Efficiency	Real-Time Monitoring	Scalability
[18]	✓	Blockchain	Limited	Partial	✗	Medium	Medium	High
[19]	✓	Blockchain	✗	✓	✗	Medium	High	Medium
[20]	✓	Blockchain, DL	Weak	✓	Moderate	✗	✓	Low
[21]	✓	Blockchain	✗	✓	✗	✗	✓	Low
[23]	✓	Blockchain	✓	✓	✓	Low	N/A	Moderate
[22]	✓	Blockchain, XAI	Limited	✓	Moderate	Low	✗	Moderate
[24]	✓	Blockchain, FL	✓	✓	✓	Low	✓	Moderate
[25]	✓	Blockchain, Auction	Limited	Moderate	Low	Low	N/A	✓
[26]	✓	Opt. FL	✓	✓	✓	Moderate	✓	✓
[27]	✗	Hybrid DL	✓	✗	✓	Moderate	✓	Medium
[28]	✗	ML	✓	✓	Partial	Medium	✓	Moderate
[29]	✗	XAI	✓	✓	✓	✓	✗	✓
[31]	✗	Honeypot, ML	High	Weak	Moderate	Low	Medium	Low
Ours	✓	Honeypot + DT + FD	High	High	High	High	High	High

**Table 2:** Performance of different AI models on the CIC IoT dataset.

AI Model	Accuracy	TPR	Precision	F1-Score
Decision Trees (DTs)	0.9708	0.8337	0.8104	0.8215
**Random Forest (RF)**	**0.9825**	**0.7955**	**0.9885**	**0.8658**
**XGBoost**	**0.9836**	**0.8195**	**0.9677**	**0.8768**
Adaboost	0.9681	0.6964	0.9599	0.7661
Logistic Regression (LR)	0.8930	0.4042	0.7874	0.4200
Quadratic Discriminant Analysis (QDA)	0.4918	0.6393	0.5262	0.4412
DNN	0.9624	0.5893	0.9156	0.6186

Note: Bold values indicate the two best-performing models across multiple evaluation metrics compared to the other models.

## Data Availability

The CIC IoT 2023 dataset used in this study is publicly available from the Canadian Institute for Cybersecurity (CIC) at: https://www.unb.ca/cic/datasets/iotdataset-2023.html (accessed on 1 July 2025).
